# Effectiveness of midwife-led brief counseling intervention on post-traumatic stress disorder, depression, and anxiety symptoms of women experiencing a traumatic childbirth: a randomized controlled trial

**DOI:** 10.1186/s12884-020-2826-1

**Published:** 2020-03-06

**Authors:** Leila Asadzadeh, Elham Jafari, Roghieh Kharaghani, Farhad Taremian

**Affiliations:** 1 Department of Midwifery, School of Nursing and Midwifery, Zanjan University of Medical Sciences, Zanjan, Iran; 2 Department of Midwifery, School of Nursing and Midwifery, Zanjan University of Medical Sciences, Zanjan, Iran; 3 Department of Midwifery, School of Nursing and Midwifery, Zanjan University of Medical Sciences, Zanjan, Iran; 4 Farhad Taremian. Substance Abuse and Dependence Research Center, University of Social Welfare and Rehabilitation Sciences, Tehran, Iran

**Keywords:** Traumatic childbirth, Post-traumatic stress disorder, Depression, Anxiety, Midwife-led brief counseling, Iran

## Abstract

**Background:**

This study investigated the effectiveness of brief midwife-led counseling based on Gamble and colleagues’ approach in decreasing post-traumatic stress disorder, depression, and anxiety symptoms among a group of women who had experienced a traumatic childbirth.

**Methods:**

From among 270 pregnant women screened to participate in the study, 90 women experienced a traumatic childbirth. They were randomly assigned into two groups: intervention (*n* = 45) and control group (*n* = 45). We did a face-to-face counseling session within 72 h after giving birth and a telephone counseling session four to 6 weeks after giving birth for the intervention group. The control group only received the postnatal routine care. The outcome measures were post-traumatic stress disorder, depression, and anxiety symptoms.

**Results:**

At the three-month follow-up, the intervention group showed significantly higher improvement on post-traumatic stress disorder, depression, and anxiety symptoms compared to the control group.

**Conclusions:**

Gamble and colleagues’ midwife-led brief counseling could be an effective approach to reduce psychological distress of women who have experienced a traumatic childbirth.

**Trial registration number:**

IRCT201608285417N2, Date of Registration: 2/21/2017.

## Background

Childbirth can be stressful for women. Researches showed that 34% [1] to 54% [2] of women have experienced childbirth as a traumatic event. This is a significant risk factor for developing post-traumatic stress disorder (PTSD) [3, 4], depression, and anxiety symptoms [5, 6].

PTSD is an anxiety disorder that occurs after a stressful event, which is characterized by an actual or potential death or a threat to the physical integrity of self or others [7]. Patients with PTSD suffer from recurrent experience of the traumatic event, avoidance, negative cognitions and emotions, irritability, hypervigilance, low concentration, and sleep disturbance [7]. It is estimated that 3.1 to 15.7% of women develop postpartum PTSD [6]. Also, women who have experienced a traumatic birth are more vulnerable to develop depression and anxiety symptoms [5, 8]. This shows the necessity of considering counseling approaches for women who are at risk of developing a psychological distress after giving birth.

Gamble and colleagues [9–11] suggested a midwife-led brief counseling intervention for postpartum women experiencing PTSD symptoms. It consists of two sessions of counseling by midwives at 48–72 h and four to 6 weeks after giving birth. This intervention emphasizes on the therapeutic relationship, acceptance of experiences, expression of emotions, reviewing labor management, increasing social support, and problem solving [10].

Effectiveness of Gamble and colleagues’ midwife-led brief counseling intervention on reducing PTSD, depression, and anxiety symptoms has been the subject of a number of studies [9, 10]. In a study, Gamble and colleagues [9] assigned at risk women into intervention and control groups. Their intervention group received two counseling sessions within 72 h and at four to 6 weeks after giving birth. This group had lower PTSD, depression, and stress symptoms at the three-month follow-up. They also had less self-blame and were more confident about future pregnancies. In another study, women who had a distressing childbirth were assigned into trauma-focused intervention or parenting advice (active control) groups during the first and sixth weeks after giving birth. The trauma-focused intervention group had significantly lower depression symptoms and better social support and adjustment to motherhood [12]. A qualitative research investigated women’s experiences of participating in midwife-led brief counseling interventions [13]. Researchers found out that women can elaborate the specific components of the intervention. The studied women mentioned that midwife-led counselling intervention has helped them to recognize the causes of their psychological distress, and to adjust better with their stress [13].

To our knowledge, only one published study has investigated the effect of counseling on post-traumatic stress disorder after a traumatic childbirth among Iranian women. In that study, Taghizadeh and colleagues [6] reported that women who participate in a face-to-face session (based on the Gamble and colleagues’ approach) within 72 h after giving birth show significantly lower PTSD symptoms than their control group at the three-month follow-up.

Considering lack of enough knowledge in this regard, we investigated the effectiveness of Gamble and colleagues’ midwife-led brief counseling intervention on reducing PTSD, depression and anxiety symptoms in a group of Iranian at-risk postpartum women.

## Method

This randomized control trial was done on pregnant women attending three governmental antenatal clinics of healthcare centers of Zanjan city, Iran. To collect data from a homogeneous group of women with low risk pregnancy, a number of inclusion and exclusion criteria were considered.

The inclusion criteria were being: 1) 18 to 35 years old, 2) able to speak and read Persian (since some women were from the less privileged parts of Zanjan province in which all people do not speak Persian and did not have enough reading and speaking language skills), 3) in the last pregnancy trimester, and 4) having a single embryo. Women were excluded if they had any of the following conditions: 1) score ≥ 10 on the Edinburgh postnatal depression scale, 2) history of abortion and infertility, 3) mental or physical chronic diseases, 4) taking medicine that causes symptoms of depression, 5) history of postpartum depression in the first-degree relatives, and 6) experience of a major stressful event during the past year.

### Sample size

Based on the mean and standard deviation of scores on the Post-Traumatic Stress Disorder Checklist-5 (PCL-5) reported for the intervention (10.1 ± 3.8) and control groups (12.5 ± 3.67) in the previous randomized clinical trial on Iranian postpartum women with PTSD (Taghizadeh, et al., 2008), power = %80, and error of type 1 = .05, the sample size of 40 was calculated for each group. Considering the attrition rate, sample size of 45 was estimated for each group.

$$ \mathrm{n}=\frac{{\left({\mathrm{Z}}_{\mathrm{a}}+{\mathrm{Z}}_{\upbeta}\right)}^2{\mathrm{S}}^2}{\Delta^2} $$

### Data collection

A number 270 pregnant women who were in the last pregnancy trimester and consecutively attended the antenatal clinics between September and November 2017 were recruited in this study. They were informed by the midwives of the clinics about this study. Those who signed a written consent were enrolled. At first, they were asked to complete a researcher-made questionnaire. This questionnaire consisted of demographic characteristics, pregnancy history, and Edinburgh postnatal depression scale before giving birth. 37 women did not meet the inclusion criteria. Therefore, 233 women were followed up until giving birth. They completed the DSM-5-A criterion for the diagnosis of traumatic childbirth scale within 72 h of giving birth. From among them, 90 women (38.62%) reported that they had experienced a traumatic birth and thus met the criterion A of DSM-5 for PTSD.

**Fig. 1:**
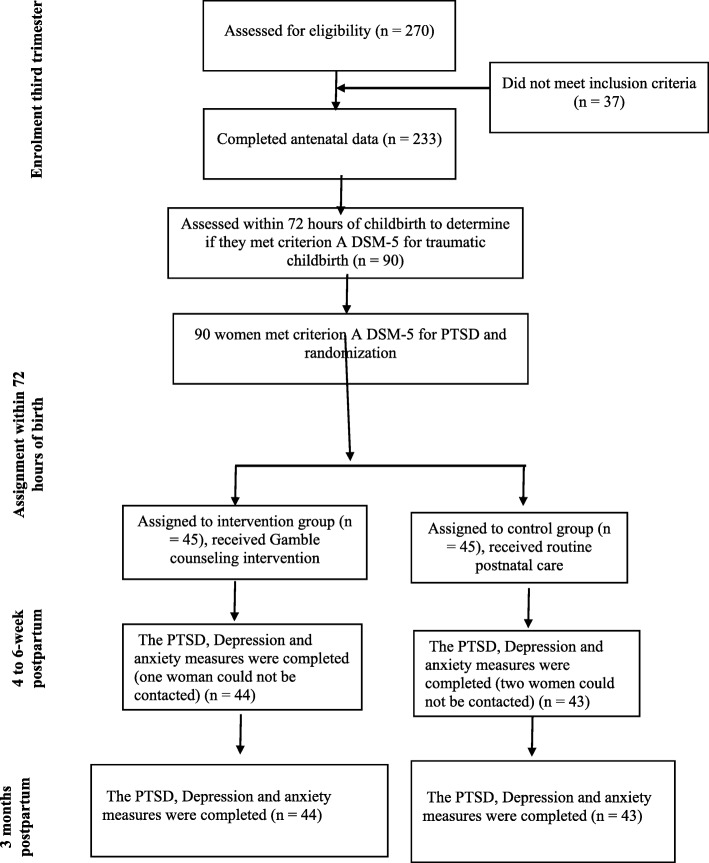
Flow diagram of the study

They were randomly assigned into two groups: the intervention (*n* = 45) or control groups (*n* = 45) via block randomization method using 4-way blocks. The randomization code was generated by a web-based randomization system. The study was double-blinded. In other words, the assessors and data analyzer were blinded to the group allocation. However, blinding was not possible for the participants and clinician. A participant in the intervention group and two participants in the control group were excluded from the study because their babies were admitted to the intensive care unit (Fig. 1). Both groups answered Edinburgh postnatal depression scale, Hamilton’s anxiety rating scale, and PCL-5 within 72 h, four to 6 weeks, and 3 months after giving birth.

*2.1.1.DSM-5-A criterion for the diagnosis of traumatic childbirth scale* consists of four questions: “Have you experienced a distressing or traumatic incident during your childbirth? Has your baby experienced a distressing or traumatic incident during birth? Have you seen a distressing or traumatic incident that happened to others during your delivery? Are you re-experiencing the memories of this distressing or traumatic incident, repeatedly?”. Sixteen psychologists and midwives assessed the content validity of the scale. The content validity ratio and index of the scale were 0.75 and 1.0, respectively. Then, 25 postpartum women completed the scale two times with a 14-day interval. The test-retest reliability and internal consistency of the scale were 0.74 and 0.85, respectively. The English version of this scale can be found in supplementary files.

*2.1.2. Edinburgh postnatal depression scale* is a 10-item self-report questionnaire that assesses postnatal depression (e.g. “I have been so unhappy that I have had difficulty sleeping”). Women were asked to answer the questions based on a four-point Likert scale [14]. The scores were from 0 to 30 and higher scores indicate severity of depression feeling. Scores above 14 ante-natal and 12 post-natal indicate depression [14]. Psychometric properties of the Persian version of Edinburgh postnatal depression scale has been established [15].

*2.1.3. Hamilton’s anxiety rating scale* is a 14-item clinical interview instrument that measures somatic and psychological symptoms of anxiety. It has been rated on a 5-point Likert scale (0 to 4) [16]. The psychometric properties of the Hamilton’s anxiety rating scale have been demonstrated in different populations [17], including Iranians [18].

*2.1.4. PCL-5* is a 20-item self-report instrument that assesses the degree to which a person has been annoyed in the past month by DSM–5 PTSD symptoms related to his or her most currently traumatic or distressing incidents based on a 5-point Likert scale (from 0 “not at all” to 4 “extremely”). The PCL-5 consists of four subscales that evaluated intrusions, avoidance, negative changes in cognition, mood, provocation and reactivity. The PCL-5 has satisfactory validity and reliability [19]. In our sample, internal consistency of the PLC-5 was 0.76.

### Procedure

The intervention group received a face-to-face counseling session by the first author within 72 h after their childbirth. After that, they had a telephone counseling session at four to 6 weeks after giving birth. Each session lasted 40 to 60 min. In addition, the intervention group could contact their midwife between the two sessions by telephone. The intervention approach was based on Gamble and colleagues’ protocol [9–11], which was translated into Persian and validated in Iran [6].

The intervention emphasized on the therapeutic relationship between the midwife and the person, accepting and working with perceptions, filling in the missing pieces, finding connections between the event and their emotions and behaviors, reviewing labor management, increasing social support, reinforcing positive approaches to coping, and exploring solutions. In our study, the fourth author, who is a professor of clinical psychology, trained and supervised the first author on how to do the interventions. The Persian version of the protocol can be retrieved from the corresponding author. The sessions were randomly recorded and listened by the fourth author to make sure that the intervention is in accordance with the principles of Gamble and colleagues’ protocol. The control group only received routine postpartum care. A midwife who was blinded to group assignment did the post-test four to 6 weeks after the women’s delivery and the three-month follow-up assessments (Fig. 1).

### Data analysis

The statistical analysis was done with the statistical package for social sciences (SPSS) software version 24. The probability value’s significance level was 0.05. The demographic characteristics of the participants were estimated with descriptive statistics. We used independent t-test and Chi-square test to compare the two groups regarding the socio-demographic characteristics. The Shapiro-Wilk test showed that the dependent variables have a normal distribution among the groups (*p* value ranged from 0.08 to 0.39). Thus, two-way repeated measure ANOVA was used to determine the differences between the two groups and also between the pretest and posttest, and follow up results on PTSD, depression and anxiety symptoms.

### Ethical considerations

The study was registered in the registry for clinical trials (IRCT201608285417N2). The ethics committee of Zanjan University of Medical Sciences approved the procedure of the research (ZUMS.REC.1395.197). All participants signed a written consent before participating in the study and they could exit at any stage of the research.

## Results

### The preliminary analysis

The participants’ means of age were 25.38 ± 4.56 and 25.62 ± 4.48 years old for the intervention and control groups, respectively. The means of their husbands’ ages were 30 ± 3.97 and 28.60 ± 4.60 years old for the intervention and control groups, respectively. The two groups were not different in terms of their own age (*t* (85) = 0.25, *p* = 0.79) and their husbands’ age (t (85) = 1.54, *p* = 0.12).

The preliminary analyses showed the two groups were not different regarding their own educational status (*x*^2^ (2, *N* = 87) = 0.82, *p* = 0.84), their husbands’ educational status (*x*^2^ (2, *N* = 87) = 2.80, *p* = 0.42), and their birth method (*x*^2^ (2, *N* = 87) = 1.29, *p* = 0.31). Similarly, they were not different regarding pre-birth scores of Edinburgh postnatal depression scale (*t* (88) = 0.40, *p* = 0.68), and PCL-5 (*t* (85) = 0.70, *p* = 0.48), Edinburgh postnatal depression scale (*t* (85) = 0.28, *p* = 0.77), and Hamilton’s anxiety rating scale (*t* (85) = 1.61, *p* = 0.11) within 72 h after birth (Table 1).

**Table 1:** Demographic characteristic of intervention group (*n* = 44), and control group (*n* = 43)

	Intervention group	Control group	*x*^2^	*p*
	N (%)	N (%)		
**Education**				
High school	12 (27.27%)	11 (25.58%)	0.82	0.84
Diploma	13 (29.54%)	16 (37.20%)		
Bachelor	18 (40.90%)	15 (34.88%)		
Master degree or higher	1(2.27%)	2 (4.65%)		
**Husbands’ education**				
High school	7 (15.90%)	13 (30.23%)	2.80	0.42
Diploma	21 (47.72%)	15 (34.88%)		
Bachelor	15 (34.09%)	15 (34.88%)		
Master degree or higher	1 (2.27%)	0 (0%)		
**Method of giving birth**				
Normal vaginal	36 (81.81%)	33 (76.74%)	1.29	0.39
Section	8 (18.18%)	10 (23.26%)		

### The intervention effects on PTSD

**Fig. 2:**
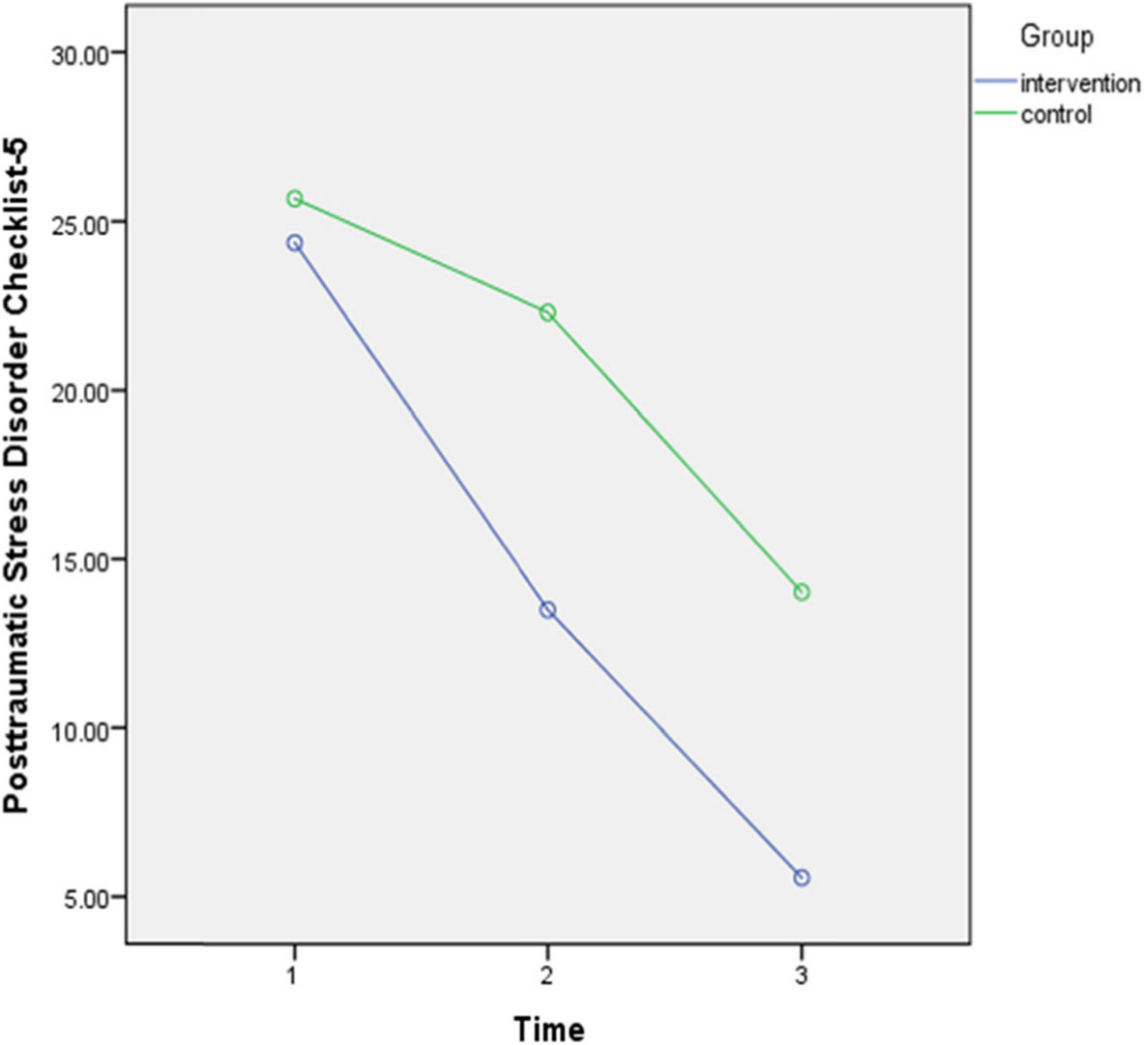
Scores of *Post-traumatic Stress Disorder Checklist-5* of intervention and control group

To confirm the effect of midwife-led counseling on PTSD symptoms, repeated measure ANOVA models tested whether the intervention group had a significant decrease in PTSD symptoms (measured by PCL-5) compared to the control group (Table 2, Fig. 2). The two groups were different in scores over time, and there was a significant interaction between the group and time factors [*f* (1,85) = 12.15, *p* = 0.0001, partial η2 = 0.12]. Also, there was a substantial main effect for time, [*f* (1,85) = 222.68, *p* = 0.0001, partial η2 = 0.72] and for group [*f* (2,57) = 24.19, *p* = 0.0001, partial η2 = 0.22].

**Table 2:** Comparison of intervention group and control group on Posttraumatic Stress Disorder Checklist-5, Edinburgh Postnatal Depression Scale, Hamilton Anxiety Rating Scale scores

	Pretest M (SD)	Posttest M (SD)	Follow up M (SD)	F (Group*time)	p
**Posttraumatic Stress Disorder Checklist-5**				12.15	0.0001
Intervention group	24.36 (8.26)	13.50 (5.88)	5.56 (5.08)		
Control group	25.67 (9.4)	22.30 (8.31)	14.02 (7.47)		
**Edinburgh Postnatal Depression Scale**				17.40	0.0001
Intervention group	7.52 (2.79)	3.43 (1.93)	1.25 (0.88)		
Control group	7.30 (4.2)	7 (3.25)	4 (1.91)		
**Hamilton Anxiety Rating Scale**				4.46	0.01
Intervention group	13.54 (5.72)	9.13 (4.25)	4.02 (2.96)		
Control group	15.69 (6.72)	15.25 (6.22)	8.51 (5.46)

The independent sample t-test indicated that the intervention group had a significantly more reduction in PCL-5 scores at four to 6 weeks (*t* (85) = 10.04, *p* = 0.0001) and 3 months postnatal (*t* (85) = 6.18, *p* = 0.0001) compared to the control group. Paired samples t-test also showed that both the intervention (*t* (43) = 10.04, *p* = 0.0001), and control groups (*t* (42) = 2.27, *p* = 0.02) had a significant decrease in PCL-5 scores at four to 6 weeks after giving birth compared to pre-test. Also, in both intervention (*t* (43) = 8.81, *p* = 0.0001) and control groups (*t* (42) = 11.77, *p* = 0.0001), PCL-5 scores significantly decreased from four to 6 weeks to 3 months postpartum. The time-group interaction indicated that the reductions were significantly more in the intervention group compared to the control group.

### The intervention effects on depression

**Fig. 3:**
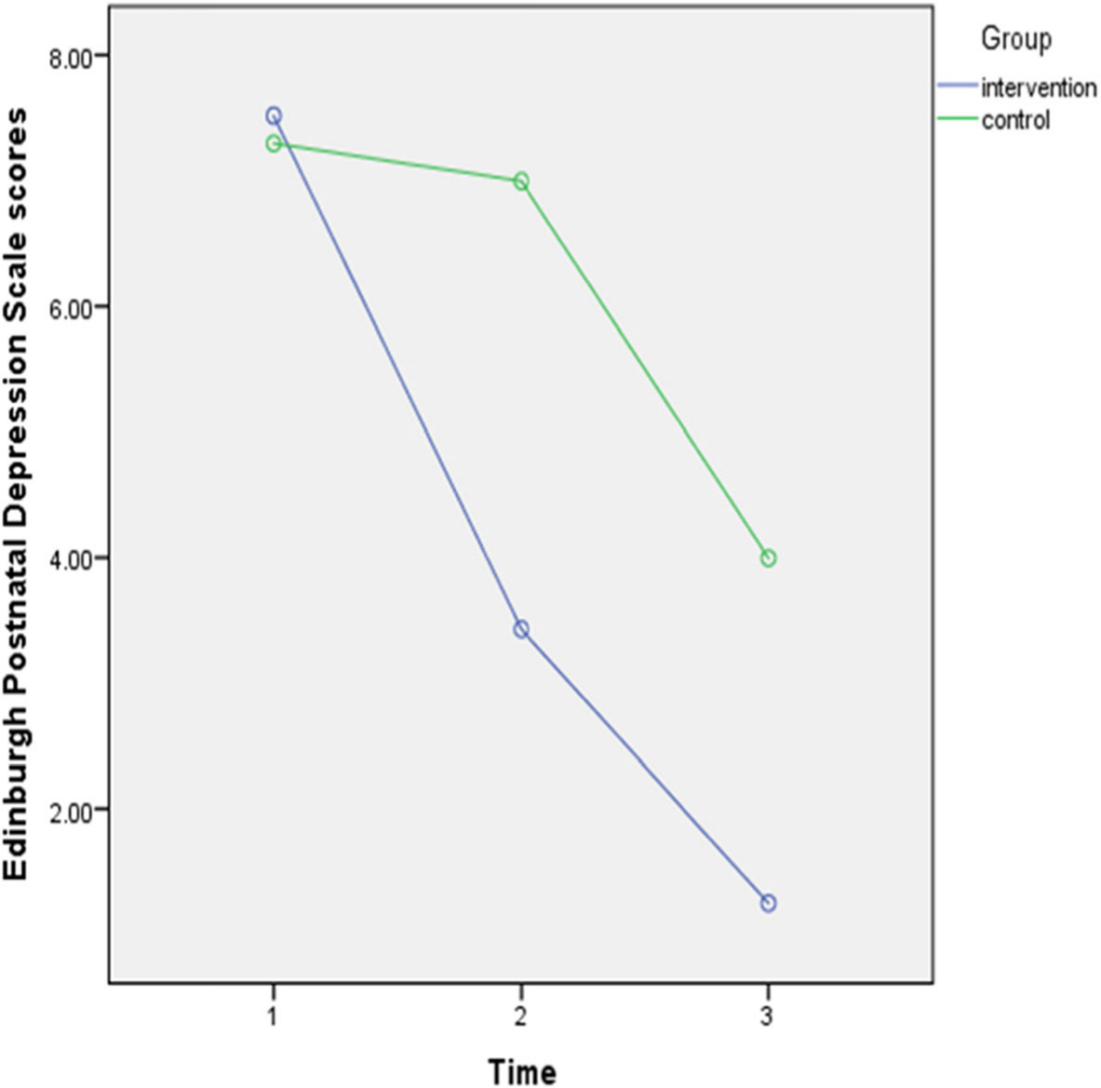
Scores of Edinburgh Postnatal Depression Scale of intervention and control group

To confirm the effect of midwife-led counseling intervention, repeated measure ANOVA models tested whether the intervention group had a significant decrease in depression symptoms (measured by Edinburgh postnatal depression scale) compared to the control group (Table 2, Fig. 3). There was a significant interaction between the group and time factors [*f* (1,85) = 17.40, *p* = 0.0001, partial η2 = 0.17]. In addition, there was a substantial main effect for time, [*f* (1,85) = 141.01, *p* = 0.0001, partial η2 = 0.62] and group [*f* (2,57) = 15.38, *p* = 0.0001, partial η2 = 0.15].

Independent sample t tests showed that the intervention group had a significantly more decrease in Edinburgh postnatal depression scale scores at four to 6 weeks (*t* (85) = 6.22, *p* = 0.0001), and 3 months after giving birth (*t* (85) = 4.61, *p* = 0.0001) compared to the control group. Also, the paired samples t-test indicated that Edinburgh postnatal depression scale scores of intervention group significantly decreased from pretest to posttest (*t* (43) = 12.92, *p* = 0.0001) and from posttest to follow-up (*t* (43) = 6.63, *p* = 0.0001). In the control group, depression scores did not decrease from pretest to posttest (*t* (42) = 0.49, *p* = 0.62), but they significantly decreased from posttest to the three-month follow up (*t* (42) = 7.78, *p* = 0.0001).

### The intervention effects on anxiety

**Fig. 4:**
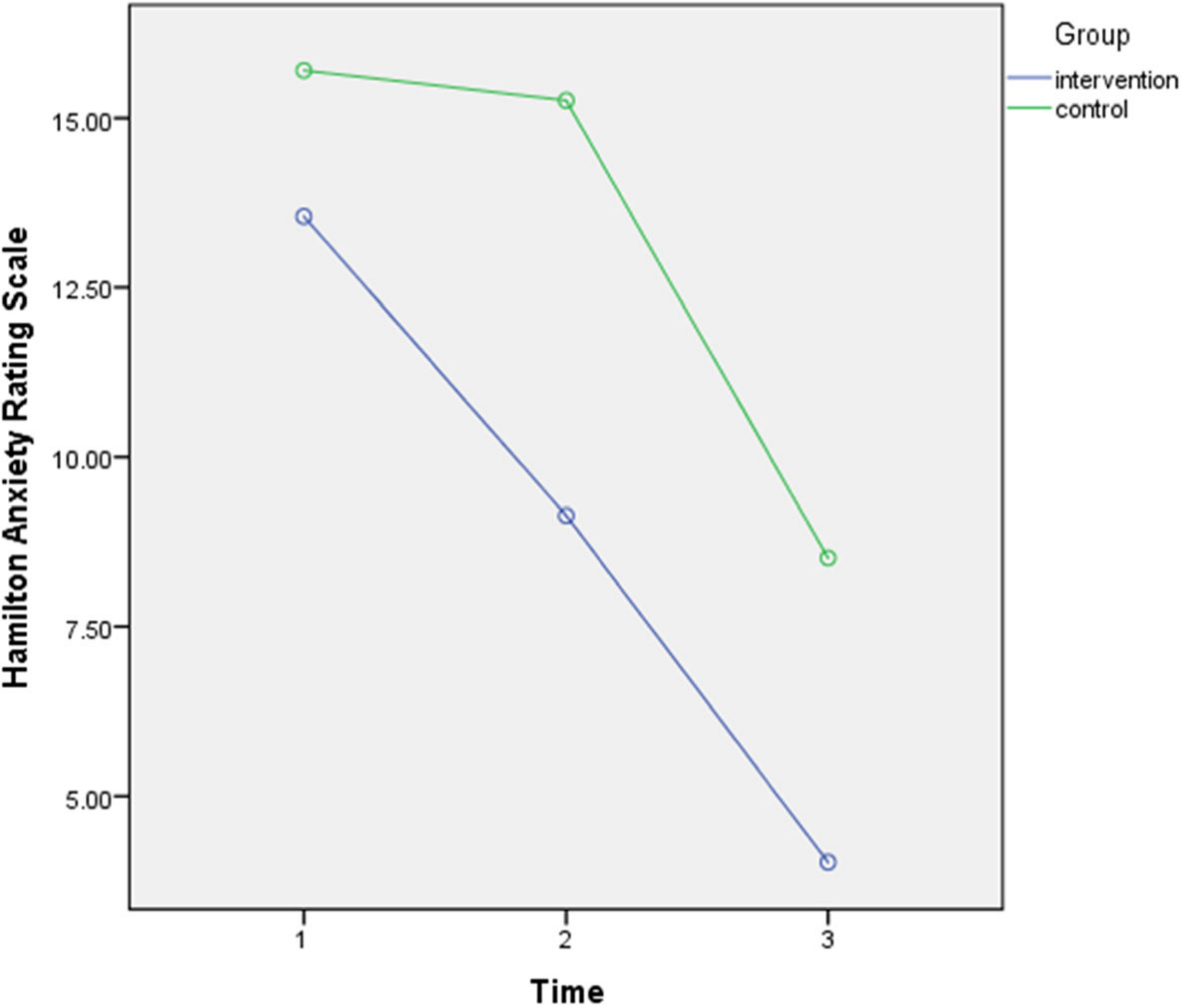
Scores of Hamilton Anxiety Rating Scale of intervention and control group

To demonstrate the effect of midwife-led counseling on anxiety symptoms, the repeated measure ANOVA models tested whether the intervention group had a significant reduction in anxiety symptoms (measured by Hamilton’s anxiety rating scale) compared to the control group (Table 2, Fig. 4). There was a significant interaction between the group and time factors [*f* (1,85) = 4.46, *p* = 0.01, partial η2 = 0.05]. Also, there was a significant main effect for time, [*f* (1,85) = 118.01, *p* = 0.0001, partial η2 = 0.58] and group [*f* (2,57) = 23.09, *p* = 0.0001, partial η2 = 0.21].

The independent sample t test showed that the intervention group had a significantly more reduction in Hamilton’s anxiety rating scale scores at four to 6 weeks (*t* (85) = 5.36, *p* = 0.0001), and 3 months postpartum (*t* (85) = 4.39, *p* = 0.0001) compared to the control group. Also, paired samples t-test indicated Hamilton’s anxiety rating scale scores of the intervention group significantly decreased from pretest to posttest (*t* (43) = 5.28, *p* = 0.0001) and from posttest to the three-month follow-up (*t* (43) = 7.63, *p* = 0.0001). In the control group, anxiety scores did not decrease from pretest to posttest (*t* (42) = 0.36, *p* = 0.72), but they significantly decreased from posttest to the three-month follow-up (*t* (42) = 10.95, *p* = 0.0001).

## Discussion

Giving birth could be a traumatic event in a women’s life. In our study, 38.62% of the studied women (*n* = 90) reported their delivery as a traumatic and distressing event. This is in line with the previous studies in which 34 to 54% of women reported their childbirth as a traumatic experience [1, 2]. Thus, at least one of three women in our study needed psychological and counseling services to overcome the negative outcomes of the traumatic childbirth. This shows the necessity of integrating psychological interventions with the routine postpartum care [9–11].

Our results show that although both groups have a significant decrease in PTSD, depression, and anxiety symptoms 3 months after giving birth, there is more decrease in the group that has had a brief counselling. Also, PTSD, depression, and anxiety symptoms significantly decreased from 72 h to four to 6 weeks and 3 months after giving birth in the intervention group. However, these symptoms did not significantly change in the control group from 72 h to four to 6 weeks postpartum. Still, the changes were significant from 72 h up to 3 months after birth. This shows that women may need more time to resolve their psychological distress after giving birth without brief counseling interventions.

The first months after giving birth are critical in the formation of the bond between the mother and her child [20]. Negative impacts of the mothers’ depression and anxiety can influence her bond with the child [21, 22]. So, it is important to reduce postpartum distress as soon as possible with effective psychological interventions. Our findings are in line with previous researches [6, 9, 23]. Still, there are studies that report that brief counseling is not significantly effective in reducing PTSD symptoms in women who suffer from traumatic childbirth [24]. Therefore, further research is needed to investigate the effectiveness of brief counseling intervention on postnatal PTSD symptoms.

Also, our results indicate that women receiving two counseling sessions had greater reduction in depression and anxiety symptoms than women who only received the routine care. These findings are consistent with previous studies that stated brief psychotherapeutic intervention is superior to routine care in terms of improvement of depression and anxiety symptoms [9, 25–27].

A positive aspect of our study was that we investigated Gamble and colleagues’ midwife-led brief counseling intervention on an Iranian population. This approach emphasizes on the counseling role of midwives in the postpartum care. It could be a logical and feasible solution to overcome the limitations for using specialized psychological and psychiatric services. Also, Gamble and colleagues’ approach focuses on increasing the social support network of women after giving birth and encourages them to express their emotions and find a connection between their cognitions, emotions, and behaviors. It uses cognitive restructuring techniques to help mothers recognize and change their dysfunctional attitudes towards childbirth and its related pain and, in turn, increase their self-efficacy and confidence for future pregnancies. Finally, it helps postpartum women to learn how to find appropriate and functional solutions for their problems.

Our study had its own advantages and limitations. The advantages were having a relatively large sample size, having a follow-up period, and measuring a variety of psychological outcomes. The limitation was that we only used self-report questionnaires to assess the outcome variables. Using structured clinical interviews helps researchers to measure variables more objectively to determine clinical significance of the intervention.

## Conclusions

Gamble and colleagues’ midwife-led brief counseling intervention could be an effective approach to reduce psychological distress of women who have experienced a traumatic childbirth. It seems important to include brief psychoeducational programs in the training of midwifery students. In addition, screening of postpartum women is recommended to identify those who have experienced a traumatic childbirth. Future researches can use structured clinical interviews to assess the effectiveness of midwife-led brief counseling intervention on mental health of postnatal women. Also, doing more studies on the effectiveness of Gamble and colleagues’ midwife-led brief counseling on the other outcomes (e.g. the bond between mother and child) is recommended.

## Abbreviations

ANOVA: Analysis of Variance
DSM-5: Diagnostic and Statistical Manual of Mental Disorders, Fifth Edition
PCL-5: Post-traumatic Stress Disorder Checklist-5
PTSD: Post Traumatic Stress Disorder
RCT: Randomized control Trail
SPSS: Statistical Package for the Social Sciences
